# Sharing data among clinical trials of therapeutics in COVID-19: Barriers and facilitators to collaborating in a crisis

**DOI:** 10.1017/cts.2021.866

**Published:** 2021-10-07

**Authors:** Marisha E. Palm, Christopher J. Lindsell, Harry P. Selker

**Affiliations:** 1 Institute for Clinical Research and Health Policy Studies, Tufts Medical Center, Boston, Massachusetts, USA; 2 Tufts Clinical and Translational Science Institute, Tufts University, Boston, Massachusetts, USA; 3 Department of Biostatistics, Vanderbilt University Medical Center, Nashville, Tennessee, USA; 4 Executive Committee members: Danny Benjamin; Gordon Bernard; Barbara Bierer; Theodora Cohen; Daniel Ford; Neil Goldenberg; Daniel Hanley; Frank Harrell; Betsy Ogburn; Daniel Scharfstein; Brian Smith

**Keywords:** COVID-19, Trial Innovation Network, CTSA, clinical trials, DSMB, data sharing, collaboration

## Abstract

**Background:**

The Clinical and Translational Science Award Program (CTSA) Trial Innovation Network (TIN) was launched in 2016 to increase the efficiency and effectiveness of multisite trials by supporting the development of national infrastructure. With the advent of the COVID-19 pandemic, it was therefore well-positioned to support clinical trial collaboration. The TIN was leveraged to support two initiatives: (1) to create and evaluate a mechanism for coordinating Data and Safety Monitoring Board (DSMB) activities among multiple ongoing trials of the same therapeutic agents, and (2) to share data across clinical trials so that smaller, likely underpowered studies, could be combined to produce meaningful and actionable data through pooled analyses. The success of these initiatives was understood to be dependent upon the willingness of investigators, study teams, and US National Institutes of Health research networks to collaborate and share information.

**Methods:**

To inform these two initiatives, we conducted semistructured interviews with members of CTSA hubs and clinical research stakeholders that probed barriers and facilitators to collaboration. Thematic analysis identified topics relevant across institutions, individuals, and DSMBs.

**Results:**

The DSMB coordination initiative was viewed as less controversial, while the data pooling initiative was seen as complex because of its potential impact on publication, authorship, and the rewards of discovery. Barriers related to resources, centralization, and technical work were significant, but interviewees suggested these could be handled by the provision of central funding and supportive frameworks. The more intractable findings were related to issues around credit and ownership of data.

**Conclusion:**

Based on our interviews, we conclude with nine recommended actions that can be implemented to support collaboration.

## Introduction

### The COVID-19 Global Pandemic

The novel coronavirus, which began in Wuhan, China and quickly spread to Europe and America, was declared a pandemic by the World Health Organization on March 11, 2019 [1]. Over a year later, the pandemic is still not under control the USA, where the death count of over 570,000 is likely an underestimate of the true impact of COVID-19 [2]. Despite warnings from the infectious disease community that a coronavirus pandemic was a looming significant threat [3], a rapid response to the unfolding global health crisis was hampered by a lack of infrastructure, organization, and coordination dedicated to tackle the challenge [4]. The clinical and translational research community has been stretched in its attempts to understand the virus and its effects and to develop prevention and treatment strategies. The necessary vaccines and treatments require testing for safety and efficacy, and this important work is being done under significant time pressure as COVID-19 continues to cause illness and death around the world. It is in these circumstances that the US National Institutes of Health (NIH) Clinical and Translational Science Award (CTSA) Program and its Trial Innovation Network (TIN) was poised, and was called on, to lead at the local, regional, and national level.

### CTSA Program Development

The CTSA program was launched by the NIH in 2006 as part of its Roadmap for Medical Research [5]. The program now supports a national network of over 60 hubs that work to speed the translational of biomedical research into patient care and improved health via training, research projects, and national initiatives [6]. In 2016, the CTSA program launched the TIN to increase the efficiency and effectiveness of multisite clinical trials by supporting the development of national infrastructure to address continued roadblocks to success [7]. The TIN is comprised of three Trial Innovations Centers (TICs), a Recruitment Innovation Center (RIC), and the CTSA hubs. The vision for the TIN is to leverage the work of the CTSA hubs to coordinate support for multisite clinical trial design, development, and operation, and to provide a platform for testing translational innovations [8]. Unlike most NIH sponsored study networks, the TIN is not tied to a particular disease or discipline. CTSA hubs each have a Liaison Team that supports collaboration and interoperability of platforms [9]. The TIN plays a role in connecting the CTSA hubs, with the goal of speeding the efficiency of large multisite clinical trials. It was, therefore, ideally positioned to support the kinds of clinical trial collaboration needed to respond in an emergency setting such as a national pandemic, supplementing the work of other research networks during a time of significant need.

### The Clinical Research Response Early in the COVID-19 Global Pandemic

Early in the pandemic, institutional and donor funding, industry funding, and federal NIH funding were provided to ongoing programs and standing networks to leverage existing research infrastructure to start COVID-19 therapeutic trials. The rapid response was not focused on cohesiveness and collaboration within and across networks. These grants and supplements led to a number of modest-sized, disconnected randomized controlled trials of promising therapeutic agents, many with different protocols and endpoints. Some of these trials were underpowered, and others struggled with recruitment as case rates waxed and waned across the country and multiple competing trials were launched.

### TIN Collaboration Efforts

In this context, the TIN Leadership identified an opportunity to leverage its existing network to support coordination among COVID-19 clinical trials. The National Center for Advancing Translational Science (NCATS), the NIH center that funds the CTSA program, and the National Institute of Neurological Disorders and Stroke provided funding to support two TIN initiatives. The first was to create and evaluate a mechanism for coordinating Data Safety and Monitoring Board (DSMB) activities among multiple ongoing trials of the same therapeutic agents. The objective was that by aggregating safety information, trials could draw conclusions based on all accrued information, including ongoing unpublished studies. The second initiative was to share data across clinical trials so that smaller, likely underpowered and thus uninformative studies, could be combined to produce meaningful and actionable data through pooled analyses. As the DSMB and pooled analysis initiatives come to completion, the details will be disseminated in the peer-reviewed literature along with the results and lessons learned from those initiatives.

The TIN leadership chose convalescent plasma to test coordination of DSMBs, given there were several large ongoing clinical trials and an unmet need for rigorous data on the efficacy of this therapy, particularly given its approval under an FDA emergency use authorization. For data pooling, the TIN selected hydroxychloroquine because there were numerous smaller trials that by themselves were unlikely to yield conclusive results. For both initiatives, success would depend on the contributions of investigators, sites, studies, and networks. However, there was no mandate for COVID DSMBs or investigators to participate and TIN leadership was operating under a model of leading without a higher directive. Therefore, to inform the implementation of these initiatives, we conducted semistructured interviews with members of CTSA hubs and clinical research stakeholders, specifically research and administrative leadership, individual investigators, statisticians and data managers, regulatory professionals, and DSMB members. This qualitative work was done in support of the two TIN initiatives. The goal was to probe the barriers and facilitators to participation in such collaborative efforts. This included inquiring about what systems and supports were needed, what personal and organizational concerns may impact decisions to collaborate, and how trust should be built and maintained. Here, we describe the results in the context of the TIN’s experience developing data pooling and DSMB coordination during a pandemic.

## Methods

We used two sources of information. The primary source was a series of semistructured interviews with clinical research stakeholders. We sampled purposively across the country to include faculty and staff, span specialties from statistics to regulatory science, and include representatives from organizations with different levels of engagement. Secondarily, we documented informal observations from developing and implementing the processes for pooling data and DSMB coordination. Observations were generated by personnel participating in the various levels of meetings from the strategy discussions of the COVID-19 initiative Executive Committee to the detailed conversations among statisticians on mapping data elements.

When identifying potential interview participants, we wanted to speak to people in institutions with different levels of engagement in COVID-19-related activities. To determine levels of engagement, we used information from the COVID Collaboration Platform, which was launched with help from the TIN, partner institutions, an executive committee, and an advisory board. A description of the two collaborative initiatives was circulated to all Hub Liaison Teams and other CTSA points of contact with a request for their “Expression of Interest.” The initiatives were also presented at the CTSA program Steering Committee and on Hub Liaison Team calls, and a survey was circulated to each of the CTSA pod leaders with a request for help identifying COVID-19 clinical trial investigators. Expressions of interest were recorded, and the level of interest (or nonresponse) was used to identify CTSA hubs spanning the range of desired engagement.

In selecting potential participants for interview, we sampled to include CTSA Principal Investigators (PIs), CTSA Executive or Managing Directors, CTSA Liaison Team leads, COVID-19 trial PIs within CTSAs, and those leading the TIN collaboration efforts. We perceived these groups, with different roles, different levels of seniority, and different perspectives, as the major players in any bottom-up, nonmandated collaboration. Four interviewees representing each of these five groups were identified from institutions across the county, ranging from institutions that were nonresponsive to the TIN call for information to those who responded rapidly with full engagement. Interviewees were chosen mainly based on the responsiveness of their institution, with the only exception being the desire to balance geography and not invite too many participants from institutions in the same state. Once we had the list of institutions, the type of interviewee was chosen simply by starting with CTSA PIs, then moving to directors, and then Liaison Team leads, then starting over with CTSA PIs.

CTSA PIs, Executive or Managing Directors, Liaison Team leads, and COVID-19 trial PIs were identified using the regularly updated Network Contacts list that is available to all who work with the TIN. COVID-19 trial PIs were identified from the Expression of Interest requests, which requested such PI’s names. All potential interviewees were sent an email to invite them to a 30-minute semistructured interview via a Zoom video conferencing platform at a time convenient to them. In total, 20 individuals from 19 of the over 60 CTSA hubs were contacted.

Interviews were led by one of the three authors. Each interview began with a brief overview of the DSMB coordination and data pooling initiatives as context, and followed a semistructured guide, shown in Table 1. Interviews were recorded and transcribed. We used the transcriptions and interview notes to conduct thematic analysis [10], generating initial themes, grouping barriers and facilitators, and then pairing those that related to the same theme.

**Table 1.** Semistructured interview schedule. DSMB, Data and Safety Monitoring Board; DUA, Data Use Agreement

CTSA Engagement Semi-Structured Interview Schedule	
Background	Do you have experience with data pooling across trials? Have you been part of a clinical trial DSMB? What does your prior experience tell you about potential challenges or benefits to coordinated collaboration? How important do you think these types of collaborative projects are or will be to public health? • Where do you think their value lies?
Barriers	If you were asked to join in a collaboration across trials, what types of things might prevent you from getting involved? • Are there organizational supports or resources that might you need in order to collaborate? • What kinds of issues might you worry about if you were asked to collaborate e.g., capacity, population, relinquishing control of data, unclear authorship? • Are there conditions that you might make that would determine your engagement in any collaboration? • Are there things that might impact how you felt about collaborating, e.g. prior commitments, impacts of collaboration on timing of results? • Would you have any concerns regarding scientific reward or incentives?
Facilitators	If you were asked to join in a collaboration across trials, what types of things would help you get involved? • Are there systems, processes or people you already have in place that might facilitate involvement? • What kinds of things would make you feel like you had trust in the collaboration process? • How do you think the ideal collaboration would look and how would it operate? • If you were part of drafting a workable DUA for a collaboration like this, what would you want to see included in it?

The work was a process improvement study to inform a collaborative initiative, and therefore it was determined not to be considered human research by Tufts Institutional Review Board (IRB). To assuage concerns that the data would be analyzed with an intent to generalize, the Vanderbilt University Medical Center IRB determined this project to pose no more than minimal risk and to be exempt from review.

## Results

### Participants

Of the 20 individuals contacted, 13 (65%) agreed to an informal, semistructured interview. The remaining seven did not respond to the initial email or the follow-up request. In two cases, the interviewee invited two additional faculty and staff members whom they perceived would have insights into the initiatives. We therefore conducted 13 interviews with 17 people. Overall, our sample included seven CTSA PIs, three Executive or Managing Directors, one CTSA Liaison Team member, five CTSA investigators, four COVID-19 trial PIs, and two COVID-19 collaboration leaders; three interviewees represented multiple stakeholders. There was no difference in responsiveness between those who were fully engaged in previous network activities, and those who were less engaged. A total of 11 out of the 20 individuals contacted had engaged with the TIN EOI; six of these agreed to be interviewed and five did not.

### Thematic Analysis

Based on our interviews, we identified four main categories of stakeholders: funders, institutions, investigators, and DSMBs. We did not directly interview funders but examined comments made by interviewees about the potential role of funding agencies. Thematic analysis identified major topics that were relevant across the key stakeholder groups. The identified themes were: personal and professional ambitions, principles, and beliefs, which we have summarized as: politics; ownership; prioritization; resources; temporal issues; technical expertise; and regulatory issues. For each theme, general barriers and facilitators to implementation were described by participants, as were specific barriers and facilitators related to the pandemic setting.

#### Institutional-level barriers and facilitators

Academic units that contribute to clinical and translational research within institutions can be small or large, and can range from individual investigators to coordinated research groups and to large university campus-wide initiatives. While these groups are diverse, they share similar interests in larger, organizational initiatives that occur at the institutional level. In Table 2, we list the institutional barriers and facilitators that interviewees identified, and how these fit within the themes identified.

**Table 2.** Institutions

	Barriers	Facilitators
Political	Struggle to agree on the same priorities within or across institutions Institutional territorialism Divisions within/between organizations	Establish committees that force hard questions to facilitate agreed prioritization Secure institutional commitment Build on existing systems and platforms
Ownership	Institutional agendas may be divergent Institutional reputation could be built or damaged	Incorporation of pan or transinstitutional governance to balance power and promote feelings of control and ownership
Priority	Competes with established institutional priorities	Requirement of data sharing by COVID funders to increase/mandate priority
Resources	Institutional resources and staff time are limited	Provision of central funding for sharing and facilitation
Temporal	Trial schedules not aligned with one another	Central frameworks provided to support alignment of trial schedules
Technical	Difficulty of aligning data elements within or between trial groups	Cross-institutional groups working together to ensure and agree sufficient data alignment
Regulatory	Tackling privacy laws, legal challenges, and requirements for IRB modification to share confidential information Additional regulatory delays causing a loss of momentum The inclusion of special populations at some institutions	Development of ways to maintain confidentiality across trials and therefore minimize privacy, legal, and IRB issues, for example, creation of a repository for sharing, providing structure and reassurance for regulators

#### Individual-level barriers and facilitators

We interviewed COVID-19 trial PIs, those overseeing clinical trial PIs as part of their administrative leadership roles, and others working on COVID-19 trial teams or within their vicinity. Each interviewee described what they perceived as the barriers and facilitators to individual investigators joining others in collaborative efforts. This information is summarized in Table 3.

**Table 3.** Investigators

	Barriers	Facilitators
Political	Territorialism Individual ego Competing investigators and/or investigator agendas Concern by investigators that they will not receive prestige, fame The feeling that sharing data may expose vulnerabilities	Build on existing systems and platforms Make people feel enfranchised Ensure investigator acknowledgement Include champions with trusted track records in the collaborative effort Provide incentives for collaborating Provide transparency re: what is offered/required when joining the collaboration
Ownership	Loss of control of a franchise, possessiveness of the data Industry or donor agendas or contracts that do not allow collaboration	Establishment of governance that supports or mandates coordination Incorporation of pan or transinstitutional governance to balance power
Priority	Competes with other investigator priorities	Requirement of data sharing by COVID funders to increase/mandate priority
Resources	Limited individual investigator, or investigator team, resources and time	Centralized funding to support and encourage sharing, facilitation
Temporal	Trial schedules not aligned with one another Data may not be synchronized across trials	Central frameworks provided to support alignment of trial schedules Provision of structure to synchronize trial data
Technical	Difficulty creating systems for making interim data available for review or pooling Requires rapid data cleaning, vetting, and auditing, as well as data harmonization and transformation	Bring together cross-institutional investigators and statisticians to agree systems for making interim data available Emphasize the benefits, for example, more power in aggregated data, that make rapid cleaning and harmonizing worthwhile
Regulatory	Securing Institutional Review Board (IRB) approval and/or revisiting consent documents to enable sharing of confidential information Potential regulatory delays causing a loss of momentum	Development of ways to maintain confidentiality across trials and therefore minimize IRB issues, for example, creation of a repository for data sharing, providing structure, and reassurance for regulators

#### DSMB-level barriers and facilitators

We asked all interviewees, some of whom had participated or were currently participating on clinical trial DSMBs, about the barriers and facilitators to DSMB coordination. We have summarized this information in Table 4.

**Table 4.** Data and Safety Monitoring Board (DSMB) coordination

	Barriers	Facilitators
Political	DSMB Territorialism DSMBs in competing networks Divisions between DSMBs with competing priorities	Involve existing, new DSMBs Secure investigator and DSMB commitment Establish committees that force hard questions to facilitate agreed prioritization
Ownership	Industry or donor agendas do not allow DSMB information sharing across trials	Incorporation of pan or transinstitutional governance to balance power across DSMBs
Priority	May compete with other ongoing DSMB priorities	COVID funders could require DSMB coordination across trials to increase priority
Resources	Limited DSMB resources and staff time DSMBs are made up of busy people who have limited bandwidth	Centralized funding for facilitated sharing across DSMBs, with development of structures that help create a shared process
Temporal	DSMB meetings may be out of sync with one another	Central frameworks to establish agreed timelines for sharing DSMB information
Technical	Difficulty of data harmonization and transformation	Crossinstitutional DSMBs working together to agree plans for harmonizing/transforming
Regulatory	Potential infringement of privacy laws when information sharing Potential legal challenges to information sharing Potential for group harms for special populations	Facilitate maintenance of confidentiality across DSMBs, for example, creation of a repository for data sharing, providing structure, and reassurance for regulators Ensure engagement of community stakeholders

### Desirability of Data Pooling and DSMB Coordination

We invited interviewees to comment on the relative desirability of the data pooling and the DSMB coordination initiatives. We emphasized that DSMB coordination would gather data from multiple ongoing trials testing a specific therapy, with the primary goal being to identify early safety and efficacy signals. These activities would respect the primacy of the individual trial, its ability to generate its own conclusions, and to publish independently. This was less controversial than the idea of data pooling for the purpose of generating a combined analysis of outcomes to draw overall inferences about efficacy and safety. However, some common themes emerged related to the need to harmonize protocols, develop data sharing methods, and agree on operational definitions. In both types of collaboration, these issues were seen to pose the same barriers, and to be helped by the same facilitators.

Barriers related to resources, centralization, and technical work were significant, but interviewees suggested these could be handled by provision of central resources, organization, and supportive frameworks. Some of the more common and more intractable findings were related to the politics of data sharing, including issues around ownership, which we elaborate on below. The data pooling initiative was viewed as a more complex undertaking because of its potential impact on publication, authorship, and the rewards of discovery. The DSMB coordination initiative, on the other hand, was viewed as more straightforward and less controversial. There was general consensus that observing safety signals earlier would be beneficial and would likely improve the safety of new therapeutics. This was seen as worth the time and resources required, especially in an emergency or pandemic scenario. Individuals commented that willingness to share data for DSMB purposes did not translate to a willingness to give up decision-making authority on their own trial, citing concerns that earlier safety and efficacy signals could lead to a trial being shut down before it has fully enrolled.

### Impact of Individual Politics, Investigator Ambitions, and Perceived Ownership

The barriers to data pooling are related to individual politics and investigator ambitions. These were described by many interviewees as being substantial, even greater than the technical and operational requirements. There was frustration that the national approach had not been coordinated, which may have led to more funding dispersal and less cohesion across trials. There were also reflections on how academic individualism might be tackled with organizational governance. Without organizational governance or a mandatory requirement, investigators’ interests in collaborating and developing a consortium may be changeable and difficult to manage. Trial and data ownership was described by interviewees as one of the largest barriers to collaboration, closely related to individual politics, but important enough to be a standalone theme. Interviewees talked about why ownership is important and what is lost (or gained) when joining a collaborative group. The fears and costs around sharing data were seen as significant.

*“What generalizes more is the concern that the individual studies have of losing their own integrity, their own independence…the splash that they might be able to make.”*

*“Fame…this is my little study…why should I share this with anyone else…why should I be the middle author…That’s the barrier. On the one hand by the PI, and of course, exactly the same thing from the sponsor. I want to be the person that funded the cure.”*

*“As a researcher …there is a cost associated with making the data sharable… It’s not that they don't want to share the data, but…They are not confident themselves in the quality of the data…you are exposed.”*

Interviewees also described barriers to data pooling related to organizational politics. There was a feeling of frustration that funders and networks were not working well together and that structures had not been established early to facilitate collaboration. Interviewees felt the lack of wider national organization around the funding and requirements for collaboration meant additional confusion and duplication. Some questioned whether it would have been helpful for collaboration to have been mandated, especially in an academic climate where competition is predominant. There was agreement across many interviewees that the systems that had been created or developed organically were not as organized as they should have been to be effective.

*“One thing that has been sort of confusing or difficult to manage during this whole crisis has been…everyone is excited to accelerate all over the place. And sometimes what that ends up looking like is chaos and so this is the CTSA network and we are keen to get things going, but… there are other networks out there and they are all doing similar things…”*

*“We did not leverage the collective intelligence and wisdom that we had in order to actually lead in this area. I think that we behaved very traditionally, and in a very single PI with a lab concept instead of a true team science concept.”*

*“Our initial efforts, and what we see of others, were not as organized as they needed to be. The global pandemic has been clearly a life altering event and therefore a consequential scientific event that deserves better organization from our research resources…”*

## Discussion

It is important to understand how to support collaboration to improve the efficiency of clinical research, especially in emergency circumstances such as a global pandemic. Although this study is limited by a small sample size, we heard from those across the country who hold a variety of different leadership and operational roles within the CTSA Program and in COVID-19 clinical trials, and we were able to identify important recurring themes. Many of the barriers to collaboration have solutions that could be addressed with resources and organizational structure. Others are more intractable and reveal issues of individual and institutional culture and reputation that remain systemic barriers to collaboration in academic medicine.

The barriers to data pooling that require resources and technical expertise are well-documented [11,12]. There is considerable work to be done to harmonize data across multiple trials, and this requires openness and concerted technical expertise. Investigators without experience in these types of efforts may underestimate the technical hurdles and the time commitment. However, those experienced in this work noted that the barriers could be overcome with the tools already at hand, particularly as the TIN had been granted supplementary funding and had brought together a centralized core of experts to tackle harmonization efforts.

Our findings suggest that desires for institutional and individual credit and prestige hinder willingness to collaborate across clinical trials. The cultural barriers related to national ethos and the competitive nature of academia have been previously documented. Academic institutions are placed in competition [13] and academics build their own empires often without the collaboration required by other types of organizations. This environment may make it difficult to create a spirit of teamwork and partnership, even when public health depends on it. Previous work has shown similar issues in academic-community collaboration for disaster preparedness, where responses were hindered by concerns about ownership of collaborative outputs [14]. Academic institutions have also failed to support global sustainability because of a mismatch between academic reward systems and structures that would need to be built to enable solution-oriented collaboration of researchers across fields [15].

Some interviewees felt that the only way to counterbalance the individual investigator ego and concerns with academic status would be for collaboration to be mandated. For example, if early funding opportunities mandated contribution to a collective, then this process might have been clearer and more streamlined. Unfortunately, in this pandemic, that may no longer be possible; therefore, collaborative efforts rely on networks that have limited funding to build centralized systems that depend on trust and prior relationships. In this context, the desire for institutional and individual credit will continue to be one of the most significant barriers to collaboration. One way to encourage data sharing is to connect it with academic reward; for example, via formal recognition of the multiplier effect that data sharing has on the global impact of a study beyond its immediate findings. Tracking and rewarding data reuse in promotion and tenure decisions could be a pathway for supporting data sharing [16,17].

Interviewees made clear the importance of central frameworks and resources, structures to support collaborative processes, and crossinstitutional bridges. Many interviewees emphasized building on existing systems and platforms within trusted networks. Indeed, this is what the TIN has been attempting, using the network of CTSA hubs, the TICs, and the RIC to support COVID-19 efforts. Although this study focused on the barriers and facilitators to the DSMB coordination and data pooling initiatives led by the TIN, many of the issues that are highlighted here are not specific to the TIN or to NIH funding. These are larger problems that play out across academic clinical research institutions and serve to hinder necessary collaborative efforts.

### Recommendations

Based on our interviews and experience, there are actions that can be taken now and/or can be implemented in the future when responding to a public health emergency. These are:

1. Build on existing networks and platforms: In the face of a public health crisis, using existing network infrastructures is suggested. There was an acknowledgement that the CTSA Program, and the TIN, are well-positioned to build on existing networks.
2. Recognize the views about the networks leading the initiative: It is important to understand the context within which initiatives are developed and maintained. The network leading the initiative must be seen as inclusive and transparent in order to build trust among a collaborative group.
3. Draw from central resources for sharing and facilitation: Depending upon distributed resources to develop a collaborative effort is ineffective. We recommend the provision of central resources to help build frameworks that support data sharing. This may include a master protocol template, shared plans for data collection, and common outcomes.
4. Establish pan- or transinstitutional governance: There is significant institutional competition within academia. Creation of distributed governance would support coordinated efforts with shared responsibility and decision-making.
5. Form oversight committees to address hard questions from a multi-stakeholder perspective: During the pandemic, committees have been created to bring network, organizational, or departmental leadership together to review COVID trials, assess their priority and make decisions about resource allocation. These have been essential to the process of asking hard questions and making important decisions, and should become a natural response to emerging issues.
6. Create a repository for sharing that is structured to maintain confidentiality: Some of the technical barriers identified by interviewees were related to the sharing of confidential data. We recommend the creation of a repository for sharing information that enables both the maintenance of confidentiality and the pooling of deidentified data as agreed upon by investigators.
7. Get existing, crossinstitutional DSMBs working together: This step, taken early in the course of the pandemic, would have diminished the need for sharing data among DSMBs and could occur in much the same way that existing networks and platforms could be leveraged for collaboration.
8. Incentivize collaboration: One of the most significant barriers to collaboration was individual or institutional competition, investigator ego, and disenfranchisement. Failure to reward collaboration is a major barrier facing multisite clinical trials. It is imperative that investigators are personally and professionally recognized for their contributions to collaborative efforts.
9. Ensure transparency around what is required and what is offered: There is a tension between the ability to be transparent and building a collaboration when the direction and scope are not yet clear. However, we recommend that the parameters of engagement and collaboration should be drawn early and revisited often.

## Conclusion

This article describes barriers and facilitators to collaboration for two data sharing initiatives undertaken during the COVID-19 pandemic. Many of the barriers have clear pathways for resolution, mostly focused on central frameworks and structures. The larger cultural and political barriers identified are generally preexisting barriers to collaboration in academia. The professional and personal reward system that emphasizes individual achievement and institutional recognition over collaboration continues, effectively, to prioritize incremental knowledge gain from small studies rather than the more significant impact that can be achieved by broad collaborations. We expect these barriers slowed the national response to the COVID-19 pandemic and would urge funders and universities to learn the important lessons that have been highlighted by the pandemic. These events and the findings we present give us an opportunity to forge a better, more collaborative path forward that serves public health and the greater good.

## References

[1] Rolling updates on coronavirus disease (COVID-19). World Health Organization , 2020, (https://www.who.int/emergencies/diseases/novel-coronavirus-2019/events-as-they-happen).

[2] Weinberger DM , Chen J , Cohen T , et al. Estimation of excess deaths associated with the COVID-19 pandemic in the United States, March to May 2020. JAMA Internal Medicine 2020; 180(10): 1336–1344. DOI 10.1001/jamainternmed.2020.3391. 32609310 PMC7330834

[3] Cheng VCC , Lau SKP , Woo PCY , Yuen KY. Severe acute respiratory syndrome coronavirus as an agent of emerging and reemerging infection. Clinical Microbiology Reviews 2007; 20(4): 660–694. DOI 10.1128/cmr.00023-07. 17934078 PMC2176051

[4] Dyer O. COVID-19: US testing ramps up as early response draws harsh criticism. BMJ 2020; 368: m1167. DOI 10.1136/bmj.m1167. 32205342

[5] NIH Launches National Consortium to Transform Clinical Research. 2006. (https://www.nih.gov/news-events/news-releases/nih-launches-national-consortium-transform-clinical-research).

[6] CTSA Program Hubs: Clinical and Translational Science Awards (CTSA) Program. National Institutes of Health . 2021. (https://ncats.nih.gov/ctsa/about/hubs).

[7] Bernard GR , Harris PA , Pulley JM , et al. A collaborative, academic approach to optimizing the national clinical research infrastructure: the first year of the Trial Innovation Network. Journal of Clinical and Translational Science 2018; 2(4): 187–192. DOI 10.1017/cts.2018.319. 31011433 PMC6474372

[8] Shah MR , Culp MA , Gersing KR , et al. Early vision for the CTSA Program Trial Innovation Network: a perspective from the National Center for Advancing Translational Sciences. Clinical and Translational Science 2017; 10(5): 311–313. DOI 10.1111/cts.12463. 28271602 PMC5593163

[9] Health NIo. CTSA Program PAR-18-940 Application Information . 2020. (https://ncats.nih.gov/ctsa/funding/par-information).

[10] Vaismoradi MJJ , Turunen H , Snelgrove S. Theme development in qualitative content analysis and thematic analysis. Journal of Nursing Education and Practice 2016; 6(5): 100–110.

[11] Blettner M , Sauerbrei W , Schlehofer B , Scheuchenpflug T , Friedenreich C. Traditional reviews, meta-analyses and pooled analyses in epidemiology. International Journal of Epidemiology 1999; 28(1): 1–9. DOI 10.1093/ije/28.1.1. 10195657

[12] Rolland B , Reid S , Stelling D , et al. Toward rigorous data harmonization in cancer epidemiology research: one approach. American Journal of Epidemiology 2015; 182(12): 1033–1038. DOI 10.1093/aje/kwv133. 26589709 PMC4675662

[13] Musselin C. New forms of competition in higher education1. Socio-Economic Review. 2018; 16(3): 657–683. DOI 10.1093/ser/mwy033.

[14] Dunlop AL , Logue KM , Vaidyanathan L , Isakov AP. Facilitators and barriers for effective academic-community collaboration for disaster preparedness and response. Journal of Public Health Management and Practice 2016; 22(3): E20–E28. DOI 10.1097/PHH.0b013e3182205087. 23238058

[15] Irwin EG , Culligan PJ , Fischer-Kowalski M , Law KL , Murtugudde R , Pfirman S. Bridging barriers to advance global sustainability. Nature Sustainability 2018; 1(7): 324–326. DOI 10.1038/s41893-018-0085-1.

[16] Bierer BE , Crosas M , Pierce HH. Data authorship as an incentive to data sharing. New England Journal of Medicine 2017; 376(17): 1684–1687. DOI 10.1056/NEJMsb1616595. 28402238

[17] Pierce HH , Dev A , Statham E , Bierer BE. Credit data generators for data reuse. Nature 2019; 570(7759): 30–32. DOI 10.1038/d41586-019-01715-4. 31164773

